# Prevalence and Residual Risk of HIV in Volunteer Blood Donors of Zhejiang Province, China, from 2018 to 2022

**DOI:** 10.1155/2024/4749097

**Published:** 2024-05-24

**Authors:** Hong Zhu, Wei Ding, Wenjuan Han, Xiaofan Zheng, Yiqing Hu, Jie Dong, Yaling Wu, Danxiao Wu, Jinhui Liu, Faming Zhu

**Affiliations:** ^1^Blood Center of Zhejiang Province, Jianye Road 789, Hangzhou, Zhejiang 310052, China; ^2^Key Laboratory of Blood Safety Research of Zhejiang Province, Jianye Road 789, Hangzhou, Zhejiang 310052, China

## Abstract

**Background:**

Blood safety levels have been significantly improved since the implementation of nucleic acid amplification technology (NAT) testing for blood donors. However, there remains a residual risk of transfusion transmission infections. This study aimed to evaluate the prevalence of HIV and its residual risk transmission among volunteer blood donors of Zhejiang Province, China, for five years after NAT implementation.

**Materials and Methods:**

All specimens and information were collected from voluntary unpaid donors at all blood services in Zhejiang Province, China, from January 2018 to December 2022. The HIV antibody or antigen and HIV RNA were detected using enzyme-linked immunosorbent assay and NAT, respectively. The HIV residual risk transmission was calculated using the incidence or window period model.

**Results:**

A total of 3,375,678 voluntary blood donors were detected, revealing an HIV prevalence of 9.92/100000. The HIV prevalence of blood donors in 12 blood services in Zhejiang Province was 6.11, 6.98, 7.45, 8.21, 8.36, 8.94, 9.04, 9.66, 9.73, 10.22, 11.80, and 12.47 per 100000 donors, without statistically significant difference observed among the services (*p* > 0.05). The HIV prevalence of males (15.49/100000) was significantly higher compared to females (1.95/100000; *p* < 0.05). There was an insignificant difference in HIV prevalence among blood donors of all different age groups (*p* > 0.05), but the HIV prevalence in the 26–35 age group and 18–25 age group was significantly higher compared to the 36–45 age group (*p* < 0.05). The difference in HIV prevalence between first-time blood donors (13.65/100,000) and repeat blood donors (6.78/100,000) was statistically significant (*p* < 0.05). From 2018 to 2022, the HIV residual risk in blood transfusion transmission was 0.266/100000.

**Conclusion:**

The prevalence of HIV among blood donors in Zhejiang Province, China, is associated with age, gender, and times of blood donation. The HIV residual risk in blood transfusion transmission remains low in the province, and increasing the rate of repeat blood donors is beneficial to improve blood safety.

## 1. Introduction

Blood transfusion therapy has important clinical significance and can be used as a treatment method or supporting means for surgical procedures, patients with hematological diseases, and obstetric bleeding [1–3]. However, the blood transfusion process also has some adverse reactions, one of which is the transfusion-transmitted infections (TTIs) caused by the pathogens [4]. Prevention of TTIs remains a key element of blood transfusion safety. Various strategies were adopted in different countries to reduce the TTI residual risk [5, 6]. The TTI residual risk primarily depends on factors such as the type of blood donation, regional pathogen prevalence, detection indicators, detection methods, and detection strategies [7–9]. In China, since 1998, voluntary blood donation has been implemented, and mandatory testing for serological indicators of hepatitis B virus (HBV), hepatitis C virus (HCV), human immunodeficiency virus (HIV), and *Treponema pallidum* (TP) has been required for blood donors [10]. Since 2016, nucleic acid amplification technology (NAT) has been used for blood screening to detect HBV DNA, HCV RNA, and HIV RNA in China [10, 11]. Therefore, the TTI residual risk has gradually decreased, and the overall level of blood transfusion safety has improved in China [12, 13].

HIV infection in TTIs remains a major concern, which largely depends on the HIV prevalence among blood donors and the characteristics of HIV-positive donors [14, 15]. According to the United Nations Acquired AIDS Programme, in 2022, approximately 39 million people were infected with HIV in the world, with 1.3 million new infections and 630000 deaths from AIDS-related diseases [16]. The Chinese government has comprehensively promoted and implemented various strategies to control the spread of HIV [17, 18]. Since 2020, the number of HIV infection cases in China has been decreasing yearly, based on data from the Center for Disease Control and Prevention, China (https://www.chinacdc.cn/). As of December 31, 2023, there were 1289700 reported cases of HIV-infected individuals or AIDS patients, with 457609 reported deaths in the Chinese mainland [19]. However, HIV prevalence rates vary significantly across different provinces and regions in China [18, 20]. In Zhejiang Province, located in eastern China, the HIV prevalence is relatively low. By the end of October 2022, there were 39452 living HIV-infected individuals in Zhejiang Province, with 4671 deaths (https://www.cdc.zj.cn/). Furthermore, of the 105 newly reported cases among students, 88.6% were linked to men who have sex with men (MSM) activities (https://www.cdc.zj.cn/). HIV can be transmitted through sexual, blood, or mother-to-child vertical transmission [18, 21, 22]. To effectively prevent HIV transmission through blood transfusion, Chinese regulations require blood donors to be tested for HIV antibodies and HIV RNA [10]. Only blood that tests negative can be used for transfusion, which helps reduce the risk of HIV transmission through blood transfusions in China.

Busch et al. reported that the HIV residual risk transmission through blood transfusion is about 1 in 2 million per unit in the Units States in 2019 [7]. However, previous studies suggest that the HIV residual risk is higher in China, with 5.4 (95% CI, 1.2–12.5) infections per million whole blood donations [23]. Shi et al. reported on the HIV infection status among blood donors in five different regions of China in 2020, indicating an HIV prevalence rate of 680.4/1 million among first-time donors based on data sourced from 2013 to 2016 [24]. Given the decreasing proportion of HIV infection among the general population since 2020 and the implementation of HIV nucleic acid testing in blood services since 2016 [10, 11], it is necessary to reassess the HIV prevalence and residual risk for blood donors in China. Therefore, we retrospectively analyzed the HIV testing data of voluntary blood donors in Zhejiang Province from January 2018 to December 2022, during which all blood donors underwent NAT for HBV DNA, HCV RNA, and HIV RNA. The distribution of HIV-positive donors among different groups of blood donors was compared based on age, gender, and the number of blood donations. Additionally, the incidence-window period model was used to evaluate the HIV residual risk transmission among blood donors in Zhejiang Province, China.

## 2. Materials and Methods

### 2.1. Blood Specimens

All study specimens were collected from voluntary unpaid donors at all blood services in Zhejiang Province from January 2018 to December 2022. There are 12 blood services distributed across 11 different municipalities, including the Zhejiang Province blood center (Hangzhou), Ningbo blood station (Ningbo), Wenzhou blood station (Wenzhou), Jiaxing blood station (Jiaxing), Huzhou blood station (Huzhou), Shaoxing blood station (Shaoxing), Jinhua blood station (Jinhua), Quzhou blood station (Quzhou), Taizhou blood station (Taizhou), Lishui blood station (Lishui), Zhoushan blood station (Zhoushan), and Yiwu blood station (Yiwu). The Jinhua blood station and Yiwu blood station are both located within the Jinhua municipality. Blood donors underwent predonation and postdonation screening following regulations formulated by the Chinese government, as previously reported [11, 24]. All specimens were collected, stored, and handled according to the manufacturer's instructions for the assays.

### 2.2. Information on the Blood Donors

All 12 blood services in Zhejiang Province use the same blood information management system, as approved by the Health Administration Department of Zhejiang Province. Therefore, this system collects information from all blood donors, including age, gender, and the number of blood donations. Donors are uniquely identified by their identification card numbers. If a donor has two or more blood donations each year, they remain counted as one blood donor for analysis.

This retrospective study was approved by the Ethical Scientific Committee of Zhejiang Provincial Blood Center, China, following the Helsinki Declaration (approval No. 2023-016).

## 3. HIV Antibody or Antigen Testing

HIV antibody or antigen screening tests were performed using enzyme-linked immunosorbent assay (ELISA) reagents from two different manufacturers. One set of reagents was from BIORAD company (Hercules, California, USA), while the other set came from various manufacturers, including Beijing Wantai Biological Pharmaceutical Company Limited (Beijing, China), Zhuhai Lizhu Reagent Company Limited (Zhuhai, China), and InTec (Xiamen, China), depending on the blood services. If any one of the reagent test results showed reactivity, the specimen was considered to have a positive reaction, following strict adherence to the manufacturer's instructions, as previously reported [11].

Specimens with positive HIV antibody reactions in the screening tests by the blood services were sent to the local Center for Disease Control and Prevention, China [10]. If the Center for Disease Control and Prevention confirmed that a person was HIV-positive or tested positive for HIV RNA, the donor was classified as HIV-positive. Otherwise, the donor was considered HIV-negative.

### 3.1. HIV RNA Testing

The HIV NAT assays were performed as described in our previous reports [11, 24]. Various manufacturers are used depending on the situation of each blood service, including 6 mini pools NAT (Roche Diagnostics, Manheim, Germany), 8 mini pools NAT (Haoyuan, Shanghai, China), and individual NAT (ID-NAT, Novartis Diagnostics, Emeryville, CA, USA). All operation procedures followed the manufacturer's instructions.

### 3.2. Statistical Analysis

The prevalence of HIV was calculated by dividing the number of confirmed HIV-positive donors (those with positive antibody or HIV RNA) in a given year by the total number of blood donors in that year, with the result expressed per 100000 donors. The HIV residual risk in blood transfusion transmission is defined as the probability of collecting blood from an asymptomatic viraemic donor infected with HIV, where the infection goes undetected by routine screening assays. It was calculated using the incidence or window-period model [25]. Here is a brief introduction. The total HIV residual risk for blood donors = residual risk in the first-time donors × the donation number of first-time donors per total number of blood donations + residual risk in the repeat donors × the donation number of repeat donors per total number of blood donations.

The calculation formula of residual risk in repeated blood donors = incidence rate × viraemic phase of the diagnostic window period (vDWP). The calculation unit is as 1/1 million blood donations. The incidence rate of repeated blood donors = number of repeat donors who tested positive during one year per total number of repeat donors in this year × 100000.

In this study, the NAT testing of donors used included 6 mini pools NAT, 8 mini pools NAT, and individual NAT. The vDWP value for the 6 or 8 mini pools NAT is unavailable in the recommendations of the World Health Organization (WHO). Therefore, we used the individual NAT vDWP value suggested by WHO for the particular virus during the period from 2018 to 2022 [25]. The window period for individual NAT of HIV RNA is 8 days. The vDWP value = window period/365.

The positive cases of first-time blood donors can result from new cases or epidemic chronic cases, and the incidence rate cannot be obtained directly. Therefore, the worst-case assumption is a three-fold incidence in first-time donors compared to the corresponding repeat donors [25].

Statistical parameter analysis was performed with the SPSS 24.0 version software, and a comparison for the rate of difference groups was performed with the Chi-square test. The *p* level of significance for each analysis was set at 0.05.

## 4. Results

### 4.1. Comparison of HIV Prevalence among Blood Donors from 2018 to 2022

From 2018 to 2022, Zhejiang Province had a total of 3375678 blood donors (Table 1). Among these, 335 donors tested positive for HIV, including 326 donors who were positive for both HIV antibody and HIV RNA and 9 donors who were HIV antibody negative but HIV RNA positive (all of whom were male).

The overall HIV prevalence among blood donors was 9.9 per 100000 donors. Table 1 shows the distribution of HIV prevalence across various blood donors from different blood services, without statistically significant differences among them (*χ*^2^ = 9.933, *p*=0.536). However, a pairwise comparative analysis revealed a significant difference between the blood station with the highest prevalence (Ningbo blood station) and the one with the lowest (Huzhou blood station) (*χ*^2^ = 4.501, *p*=0.034). The HIV prevalence among different blood services has fluctuated over the years.

### 4.2. HIV Prevalence in Donors by Gender

In all blood services, the HIV prevalence among male donors was higher than that among females. The overall HIV prevalence was 15.49/100000 for males and 1.95/100000 for females (Table 2). The HIV prevalence among males was significantly higher than among females (*χ*^2^ = 151.020, *p* < 0.001).

### 4.3. HIV Prevalence in Donors with Different Ages

Overall, there was an insignificant difference in HIV prevalence among blood donors of different ages (*χ*^2^ = 8.334, *p*=0.080). However, the HIV prevalence among donors aged 26–35 and those aged 18–25 was significantly higher than among donors aged 36–45 (*χ*^2^ = 5.923, *p*=0.015; *χ*^2^ = 5.656, *p*=0.017).

### 4.4. HIV Prevalence in First-Time and Repeat Blood Donors

Except for the Zhoushan blood station, the HIV prevalence among first-time blood donors was higher than that of repeat blood donors. The overall HIV prevalence was 13.65/100,000 for first-time blood donors and 6.78/100,000 for repeat donors (Table 2), with a statistically significant difference between the two groups (*χ*^2^ = 39.916, *p* < 0.001).

### 4.5. HIV Transmission Residual Risk Estimates

From 2018 to 2022, the HIV residual risk in blood transfusion transmission was 0.266/100000 (Table 3).

## 5. Discussion

Detecting indicators of HIV infection in blood donors is a crucial step in preventing HIV transmission through blood transfusion. Currently, HIV antibodies/p24 antigens (HIV Ag/Ab1 + 2) tests and HIV RNA tests are mandatory for blood donors in China, ensuring blood safety [10]. While serology tests and NAT are performed in the same blood services in China, Zhejiang Province has implemented centralized NAT since 2016 [11, 26]. The samples of the blood donors from the original 12 blood services were centralized for NAT in four blood services (Hangzhou, Ningbo, Jinhua, and Wenzhou) [11]. Additionally, a unified blood information management system has been established for all blood services, facilitating comprehensive analysis of all blood donors' information in the Zhejiang Province [11, 26, 27]. This study retrospectively analyzes the HIV testing results of all blood donors in the Zhejiang Province over the past 5 years based on a large size of samples using the blood information management system, which will help improve blood donor recruitment strategy and HIV prevention and control strategies.

At the end of 2021, the total number of people infected with HIV in the Chinese mainland was approximately 1.038 million (https://weekly.chinacdc.cn/). Among them, 77.4% were men, and 22.6% were women. The proportion of male donors was significantly higher than that of female donors in our study, consistent with the general population [20]. However, the HIV infection rate among blood donors in Zhejiang Province was significantly lower than that among the Chinese mainland donors in the previous report [23, 24]. According to the 5-year data in our study, the HIV infection rate of blood donors in Zhejiang Province is demonstrating a downward trend. This may be attributed to continuous measures taken by the blood services in Zhejiang Province to prevent the spread of HIV in recent years. Blood services can effectively reduce the number of HIV-infected individuals among blood donors through measures such as improving public awareness and understanding of blood donation and blood safety, as well as self-exclusion of blood donors [28–30]. Other contributing factors include the low HIV prevalence in Zhejiang Province and a decreasing HIV infection rate among the general population since 2020 (https://www.cdc.zj.cn/). It was reported that new HIV infections decreased by 11.4% from January to October 2022 in Zhejiang Province compared to the same period in 2021 (https://www.cdc.zj.cn/).

Previous studies have reported that the HIV infection rate among first-time blood donors is significantly higher than that among repeat blood donors [24, 31], a viewpoint supported by our data. Therefore, from a blood safety perspective, it is necessary to strengthen the retention of blood donors and encourage them to become regular donors as much as possible, thereby increasing the rate of repeat donors, which will benefit blood safety. It has been reported that the age distribution of HIV-infected individuals in China is continuously changing [32, 33]. Currently, the incidence of new HIV diagnoses among individuals aged 60  years and older has rapidly increased, posing a significant challenge in China [32]. In our study, HIV prevalence was relatively high among blood donors aged 18–25 and 26–35 groups. Additionally, nine male specimens showed HIV antibody negative and HIV nucleic acid positive. Although we did not track these specimens, it may primarily be due to MSM transmission based on previous similar data [34, 35]. Currently, the proportion of HIV infections among MSM is increasing in China, especially among young students [34, 35]. Research on reducing this MSM risk through publicity and education is worthy of consideration for the future.

Although HIV infectious markers are routinely tested for blood donors, several factors can affect the accuracy of HIV testing, such as window period and technical limitations [7]. Therefore, there remains an HIV residual risk in blood transfusion transmission [7–9]. This residual risk value is commonly used as a monitoring indicator of blood safety in blood services. Studies have indicated that the HIV residual risk in blood transfusion transmission varies across different countries and regions [7–9]. Our study's data showed that the HIV residual risk in blood transfusion transmission in Zhejiang Province is lower than the standard for the prevention of transfusion-related HIV transmission risk set by the WHO (less than 0.5 cases per 1 million units of blood transfusion). Furthermore, the HIV residual risk in our study is significantly lower than that reported previously for the Chinese population [23], indicating a decrease in the HIV residual risk in blood transfusion transmission in China due to effective management measures. Notably, there are significant differences in HIV prevalence among different regions, resulting in various HIV residual risks across China.

In conclusion, we conducted an analysis of the HIV prevalence rate among all blood services in Zhejiang Province, China, which has implemented HIV nucleic acid testing in blood services since 2016. We also analyzed the association of HIV prevalence among blood donors with age, gender, and frequency of blood donation. Based on the data, the HIV residual risk in blood transfusion transmission is relatively low in Zhejiang Province, indicating the effectiveness of the blood testing strategy we employed in ensuring blood safety. Therefore, it is necessary to maintain and consolidate the implemented testing strategy to continuously improve blood safety [36].

## Figures and Tables

**Table 1:** The HIV prevalence of blood donors from different blood services in Zhejiang Province, China.

Blood services^*∗*^	Huzhou	Quzhou	Zhoushan	Yiwu	Jiaxing	Jinhua	Lishui	Taizhou	Shaoxing	Hangzhou	Wenzhou	Ningbo	Total
Number of positives	10	9	5	6	23	26	15	23	23	83	57	55	335
Number of donors	163796	128929	67122	73120	274983	290770	165865	238159	236455	812132	483211	441136	3375678
Prevalence^#^	6.11	6.98	7.45	8.21	8.36	8.94	9.04	9.66	9.73	10.22	11.80	12.47	9.92

^*∗*^Huzhou: blood station of Huzhou; Quzhou: blood station of Quzhou; Zhoushan: blood station of Zhoushan; Yiwu: blood station of Yiwu; Jiaxing: blood station of Jiaxing; Jinhua: blood station of Jinhua; Lishui: blood station of Lishui; Taizhou: blood station of Taizhou; Shaoxing: blood station of Shaoxing; Hangzhou: the blood center of Zhejiang Province; Wenzhou: blood station of Wenzhou; Ningbo: blood station of Ningbo. ^#^Prevalence: number per 100,000 donors.

**Table 2:** Prevalence of HIV in the donors by gender, age, and number of donations.

Variable		Hangzhou			Wenzhou			Ningbo			Jinghua		
		N	Donors	Prevalence (95% CI)	N	Donors	Prevalence (95% CI)	N	Donors	Prevalence (95% CI)	N	Donors	Prevalence (95% CI)

Gender	Men	76	477773	15.91 (12.33–19.48)	52	290223	17.92 (13.05–22.78)	52	260808	19.94 (14.52–25.35)	25	165799	15.08 (9.17–20.99)
	Women	7	334359	2.09 (0.54–3.64)	5	192988	2.59 (0.32–4.86)	3	180328	1.66 (−0.22–3.55)	1	124971	0.8 (−0.77–2.37)
Age	18–25	33	310785	10.62 (7.00–14.24)	19	123324	15.41 (8.48–22.33)	19	126746	14.99 (8.25–21.73)	11	87860	12.52 (5.12–19.92)
	26–35	27	224408	12.03 (7.49–16.57)	22	156510	14.06 (8.18–19.93)	20	147069	13.6 (7.64–19.56)	4	67619	5.92 (0.12–11.71)
	36–45	12	171051	7.02 (3.05–10.98)	9	136797	6.58 (2.28–10.88)	11	109326	10.06 (4.12–16.01)	6	73714	8.14 (1.63–14.65)
	46–55	10	99554	10.04 (3.82–16.27)	7	65164	10.74 (2.78–18.70)	5	55642	8.99 (1.11–16.86)	4	56712	7.05 (0.14–13.96)
	>55	1	6334	15.79 (−15.16–46.73)	0	1416	0	0	2353	0	1	4865	20.55 (−19.74–60.84)
Number of donations	1	54	400120	13.5 (9.9–17.09)	41	237273	17.28 (11.99–22.57)	36	206895	17.4 (11.72–23.08)	14	128234	10.92 (5.2–16.63)
	≧2	29	412012	7.04 (4.48–9.60)	16	245938	6.51 (3.32–9.69)	19	234241	8.11 (4.46–11.76)	12	162536	7.38 (3.21–11.56)

Variable		Jinghua			Taizhou			Jiaxing			Lishui		
		N	Donors	Prevalence (95% CI)	N	Donors	Prevalence (95% CI)	N	Donors	Prevalence (95% CI)	N	Donors	Prevalence (95% CI)

Gender	Men	25	165799	15.08 (9.17–20.99)	16	137186	11.66 (5.95–17.38)	23	166158	13.84 (8.18–19.50)	14	99652	14.05 (6.69–21.41)
	Women	1	124971	0.80 (−0.77–2.37)	7	100973	6.93 (1.8–12.07)	0	108825	0	1	66213	1.51 (−1.45–4.47)

Age	18–25	11	87860	12.52 (5.12–19.92)	3	44162	6.79 (−0.89–14.48)	4	75285	5.31 (0.11–10.52)	3	37002	8.11 (−1.07–17.28)
	26–35	4	67619	5.92 (0.12–11.71)	8	62207	12.86 (3.95–21.77)	12	95861	12.52 (5.44–19.60)	6	39570	15.16 (3.03–27.29)
	36–45	6	73714	8.14 (1.63–14.65)	7	72924	9.60 (2.49–16.71)	6	65536	9.16 (1.83–16.48)	3	47015	6.38 (−0.84–13.60)
	46–55	4	56712	7.05 (0.14–13.96)	4	55482	7.21 (0.14–14.27)	1	36309	2.75 (−2.64–8.15)	3	40043	7.49 (−0.99–15.97)
	>55	1	4865	20.55 (−19.74–60.84)	1	3384	29.55 (−28.38–87.46)	0	1992	0	0	2235	0

Number of donations	1	14	128234	10.92 (5.20–16.63)	16	96677	16.55 (8.44–24.66)	15	134167	11.18 (5.52–16.84)	7	58547	11.96 (3.10–20.81)
	≧2	12	162536	7.38 (3.21–11.56)	7	141482	4.95 (1.28–8.61)	8	140816	5.68 (1.74–9.62)	8	107318	7.45 (2.29–12.62)

Variable		Shaoxing			Quzhou			Huzhou			Zhoushan		
		N	Donors	Prevalence (95% CI)	N	Donors	Prevalence (95% CI)	N	Donors	Prevalence (95% CI)	N	Donors	Prevalence (95% CI)

Gender	Men	23	136526	16.85 (9.96–23.73)	7	75453	9.28 (2.41–16.15)	10	95827	10.44 (3.97–16.9)	4	41364	9.67 (0.19–19.15)
	Women	0	99929	0	2	53476	3.74 (−1.44–8.92)	0	67969	0	1	25758	3.88 (−3.73–11.49)

Age	18–25	8	76632	10.44 (3.21–17.67)	3	31547	9.51 (−1.25–20.27)	5	47380	10.55 (1.30–19.80)	2	24518	8.16 (−3.15–19.46)
	26–35	5	60338	8.29 (1.02–15.55)	0	31190	0	4	51578	7.76 (0.16–15.35)	0	19161	0
	36–45	4	57716	6.93 (0.14–13.72)	3	34578	8.68 (−1.14–18.49)	1	39837	2.51 (−2.41–7.43)	2	14592	13.71 (−5.29–32.70)
	46–55	6	39124	15.34 (3.07–27.60)	3	29234	10.26 (−1.35–21.87)	0	23642	0	1	8296	12.05 (−11.57–35.68)
	>55	0	2645	0	0	2380	0	0	1359	0	0	555	0

Number of donations	1	12	97941	12.25 (5.32–19.18)	4	49029	8.16 (0.16–16.15)	7	71359	9.81 (2.54–17.08)	1	30684	3.26 (−3.13–9.65)
	≧2	11	138514	7.94 (3.25–12.63)	5	79900	6.26 (0.77–11.74)	3	92437	3.25 (−0.43–6.92)	4	36438	10.98 (0.22–21.73)

Variable		Yiwu			Total								
		N	Donors	Prevalence (95% CI)	N	Donors	Prevalence (95% CI)						

Gender	Men	6	41775	14.36 (2.87–25.85)	308	1988544	15.49 (13.76–17.22)						
	Women	0	31345	0	27	1387134	1.95 (1.21–2.68)						

Age	18–25	2	18820	10.63 (−4.10–25.35)	112	1004061	11.15 (9.09–13.22)						
	26–35	2	21195	9.44 (−3.64–22.51)	110	976706	11.26 (9.16–13.37)						
	36–45	1	19557	5.11 (−4.91–15.14)	65	842643	7.71 (5.84–9.59)						
	46–55	1	12663	7.90 (−7.58–23.38)	45	521865	8.62 (6.10–11.14)						
	>55	0	885	0	3	30403	9.87 (−1.30–21.03)						

Number of donations	1	4	34734	11.52 (0.23–22.80)	211	1545660	13.65 (11.81–15.49)						
	≧2	2	38386	5.21 (−2.01–12.43)	124	1830018	6.78 (5.58–7.97)						

Number of HIV-positive donors; Huzhou: blood station of Huzhou; Quzhou: blood station of Quzhou; Zhoushan: blood station of Zhoushan; Yiwu: blood station of Yiwu; Jiaxing: blood station of Jiaxing; Jinhua: blood station of Jinhua; Lishui: blood station of Lishui; Taizhou: blood station of Taizhou; Shaoxing: blood station of Shaoxing; Hangzhou: the blood center of Zhejiang Province; Wenzhou: blood station of Wenzhou; Ningbo: blood station of Ningbo. Prevalence: number per 100,000 donors.

**Table 3:** HIV residual risk in blood transfusion transmission.

Donor type	Donors	Donations	Positive	Incidence^a^ (1/100,000)	vDWP^b^	Residual risk	N^#^	Overall residual risk (95% CI) (1/1000,000)
First-time donors	1545660	1545660	211	N/A	0.0219	0.444	6.863	0.266 (0.219–0.313)
Repeat donors	1830018	2349472	124	6.77	0.0219	0.148	3.477	

Viraemic phase of the diagnostic window period (vDWP); confidence interval (CI); Number of vDWP blood donations. ^a^N/A means no data. The positive cases of first-time blood donors can come from new cases or epidemic chronic cases, and the incidence rate cannot be obtained directly. ^b^The window period for individual NAT of HIV RNA is 8 days. The vDWP value = window period/365 = 8/365 = 0.0219. ^#^Number (N) of vDWP blood donations from repeat donors = residual risk × total number of donations from repeat donors/1000000; number (N) of vDWP blood donations from first-time donors = residual risk × total number of donations from first-time donors/1 000,000.

## Data Availability

The data used in this study are available from the corresponding author upon reasonable request.
